# MiR-148a Functions as a Tumor Suppressor by Targeting CCK-BR via Inactivating STAT3 and Akt in Human Gastric Cancer

**DOI:** 10.1371/journal.pone.0158961

**Published:** 2016-08-12

**Authors:** Beiqin Yu, Xin Lv, Liping Su, Jianfang Li, Yingyan Yu, Qinlong Gu, Min Yan, Zhenggang Zhu, Bingya Liu

**Affiliations:** 1 Shanghai Key Laboratory of Gastric Neoplasms, Shanghai Institute of Digestive Surgery, Ruijin Hospital, Shanghai Jiao Tong University School of Medicine, Shanghai, China; 2 Department of Vascular Surgery, Shanghai Ninth People's Hospital, Shanghai Jiao Tong University School of Medicine, Shanghai, China; The University of Texas MD Anderson Cancer Center, UNITED STATES

## Abstract

MicroRNAs (miRNAs) have been widely accepted as a class of gene expression regulators which post-translationally regulate protein expression. These small noncoding RNAs have been proved closely involved in the modulation of various pathobiological processes in cancer. In this research, we demonstrated that miR-148a expression was significantly down-regulated in gastric cancer tissues in comparison with the matched normal mucosal tissues, and its expression was statistically associated with lymph node metastasis. Ectopic expression of miR-148a inhibited tumor cell proliferation and migration *in vitro*, and inhibited tumor formation *in vivo*. Subsequently, we identified cholecystokinin B receptor (CCK-BR) as a direct target of miR-148a using western blot and luciferase activity assay. More importantly, siRNA-induced knockdown of CCK-BR elicited similar anti-oncogenic effects (decreased proliferation and migration) as those induced by enforced miR-148a expression. We also found that miR-148a-mediated anti-cancer effects are dependent on the inhibition of STAT3 and Akt activation, which subsequently regulates the pathways involved in cell proliferation and migration. Taken together, our results suggest that miR-148a serves as a tumor suppressor in human gastric carcinogenesis by targeting CCK-BR via inactivating STAT3 and Akt.

## Introduction

Gastric cancer, as one of the most common digestive malignant tumors, is a leading cause of cancer-related death worldwide [1]. To date, the long-term prognosis of patients with advanced gastric cancer hasn’t been largely improved. The main reason is an insufficient understanding of the mechanism underlying gastric carcinogenesis and a lack of biomarkers for early diagnosis. Therefore, achieving a better understanding of the molecular mechanism underlying gastric carcinogenesis might improve the diagnosis, prognosis and treatment of gastric cancer.

MiRNAs are evolutionarily conserved, endogenous non-coding single-stranded RNAs about 20–23 nucleotides in length. MiRNAs negatively regulate protein expression through complementarity between the miRNA seed sequence and the 3’ untranslated region (3’UTR) of its target gene, resulting in degradation of target mRNAs and/or inhibition of mRNAs translation [2]. As a large family of gene regulators, miRNAs are closely associated with various cellular processes, such as proliferation, apoptosis, differentiation and metabolism [3]. These cellular processes are commonly found dysregulated in cancer, suggesting that miRNAs may be involved in carcinogenesis. MiRNAs are found aberrantly expressed in a variety of human malignancies, such as lung, prostate, pancreas, liver, kidney and esophagus cancers [4–9].

By comparing miRNA expression profiles, we found that miR-148a was dramatically down-regulated in gastric cancer tissues compared with the matched normal mucosal tissues. Dysregulation of miR-148a was also reported in chronic lymphocytic leukemia, hepatoblastoma, breast cancer, cholangiocarcinoma and gastric cancer [10–14]. In the current study, we detected miR-148a expression in gastric cancer tissues and the matched normal mucosal tissues using quantitative real-time PCR (qRT-PCR), and analyzed the correlation between miR-148a expression and the clinicopathologic features. Subsequently, we embarked a comprehensive study of miR-148a in gastric cancer and demonstrated that miR-148a exerts anti-oncogenic effects *in vitro and in vivo*. CCK-BR, as a member of G-protein coupled receptors for gastrin and cholecystokinin, is principally expressed in the central nervous system and the gastrointestinal tract and mainly involved in gastric acid secretion and calcium signaling pathway. Using bioinformatics and experimental method, CCK-BR was mechanistically identified as a direct target of miR-148a. In conclusion, our results suggest that miR-148a functions as a gastric cancer suppressor through regulation of CCK-BR and its downstream effectors.

## Materials and Methods

### Ethics Statement

Written informed consent in the study was obtained from all patients. This study was approved by the ethics committee of Ruijin Hospital, Shanghai Jiao Tong University School of Medicine. Animal procedures were carried out according to a protocol approved by the Institutional Animal Care and Use Committee (IACUC) of Ruijin Hospital, Shanghai Jiao Tong University School of Medicine.

### Tissue Samples

Primary gastric cancer tissues and matched normal mucosal tissues were collected from 73 gastric cancer patients undergoing radical gastrostomy at the Department of Surgery, Ruijin Hospital, Shanghai Jiao Tong University School of Medicine. None of the patients undergoing surgery received preoperative treatment. Tissue samples were immediately snap-frozen in liquid nitrogen and stored in a refrigerator at −80°C. Clinicopathological data were reviewed, and TNM staging classification was based on criteria of American Joint Committee on Cancer (AJCC, 6th edition). All samples were verified by pathological examination.

### Cell Lines and Culture

Gastric cancer cell lines SNU-1 (ATCC: CRL-5971), SNU-16 (ATCC: CRL-5974), AGS (ATCC: CRL-1739), NCI-N87 (ATCC: CRL-5822) and KATOIII (ATCC: HTB-103) were purchased from the American Type Culture Collection (Manassas, VA, USA). Gastric cancer cell lines SGC-7901, BGC-823, MKN-45 and MKN-28 were purchased from Shanghai Institutes for Biological Sciences, Chinese Academy of Science (Shanghai, China). The human embryonic kidney cell line 293T (HEK 293T) was preserved in our institute. The gastric cancer cell lines were routinely cultured in RPMI 1640 supplemented with 10% heat-inactivated fetal bovine serum (FBS). HEK 293T was cultured in DEME supplemented with 10% heat-inactivated FBS. Exponentially growing cells were used for experiments.

### MicroRNA microarray

Twenty-eight pairs of gastric cancer tissues and the matched normal mucosal tissues were performed by human microRNA microarray v.12.0 (Agilent Technologies). The procedure and images process method as described previously [15].

### RNA Isolation and qRT-PCR

Total RNA isolation from tissues or cell lines was performed using Trizol reagent (Invitrogen, Carlsbad, CA, USA) according to the manufacturer’s instructions. The expression level of mature miR-148a in cell lines and tissue samples was detected by qRT-PCR and calculated as described [16]. The expression level of CCK-BR mRNA was measured by qRT-PCR according to the Taqman® Gene Expression Assays (Applied Biosystems, Foster City, CA, USA). The GAPDH mRNA level was used for normalization. The relative expression of CCK-BR mRNA compared with GAPDH mRNA was calculated using the 2^-ΔCT^ method.

### RNA Oligonucleotides and Transfection

MiR-148a mimics and negative control, miR-148a inhibitors and negative control were purchased from GenePharma (Shanghai, China). The specific siRNA sequence 5’-AAGCGCGTGGTGCGAATGTTG-3’ resides in exon 5 of the human CCK-BR gene (Genbank accession No. NM_176875). The control siRNA sequence 5’-AAGCTTCATAAGGCGCATAGC-3’ is located on chromosome 11 of the mouse and has no homology with the human genome by BLAST comparison. Transfection procedure as described in detail previously [17].

### Cell Proliferation Assay

SGC-7901 and MKN-45 cell proliferation was accessed by Cell Counting Kit-8 (CCK-8; Dojindo, Kumamoto, Japan) according to the manufacturer’s instructions. At 24 h post-transfection with miRNA inhibitors or inhibitor control, SGC-7901 or MKN-45 cells were seeded into 96-well plates (3 × 10^3^ cells/well), and cell proliferation was monitored as described previously [17]. After construction of miR-148a expression vector (p*Silencer*/miR-148a) and selection of stably transfected NCI-N87 cells, cell proliferation assay was performed in the same way. All experiments were performed in triplicate.

### Cell Migration Assay

Cell migration of NCI-N87, SGC-7901 and MKN-45 was assessed using Transwell chambers (8 μm, 24-well format; Corning, NY, USA) as described in detail previously [17]. Five randomly selected fields with 100× magnification were taken and counted to minimize the bias and the data were shown as the mean ± S.D.

### Construction of miR-148a Expression Vector

The 479 bp DNA fragment encoding the pre-miR-148a hairpin and flanking sequences was PCR-amplified using SGC-7901 cell genomic DNA as the template with the following primers: 5’-ACCCGCTTCAAGGGAATTGGT-3’ and 5’-TTGCTGTGACATTGCGACCAG-3’ and cloned into the BamHI and HindIII sites of the p*Silencer* 4.1-CMV hygro vector (Applied Biosystems). The primers were synthesized by Sangon Biotech (Shanghai, China) and the construct (p*Silencer*/miR-148a) was verified by sequencing.

### Stable Transfection of miR-148a Expression Vector

NCI-N87 cells were seeded into 6-well plates and transfected with p*Silencer*/miR-148a or p*Silencer*/nc vector. G418 (800 mg/l; Sigma Chemical, St Louis, MO, USA) containing medium was used for selection for three weeks and two stably transfected cell clones named as NCI-N87/miR-148a and NCI-N87/nc were chosen and maintained in medium containing 400 mg/l G418 for further study. Using qRT-PCR, the miR-148a expression in NCI-N87/miR-148a cells was proved dramatically up-regulated compared with that in NCI-N87/nc and parent NCI-N87 cells.

### Tumor Xenograft Model

A volume of 100 μl PBS containing 1×10^6^ cells of NCI-N87/miR-148a, NCI-N87/nc or NCI-N87 was injected into the right flank region of 4-week-old male nude mice (Institute of Zoology, Chinese Academy of Sciences, Shanghai, China), which were housed at a specific pathogen-free environment. Basing on our experience, the doubling time of NCI-N87 cells is about 4 days and tumor volume in control group reaches to 800–1000 mm^3^ at 4 weeks post-injection. Thus, tumor nodules were measured every 4 days. Meanwhile, aspects of healthy condition, such as body weight, skin color and mobility were evaluated. Tumor volume was evaluated using the following formula: volume = 1/2 × length × width^2^. Reduced mobility was observed in the mice of NCI-N87 group, but no mouse died. Mice were euthanized by CO_2_ asphyxiation after 4 weeks and the tumors were harvested, measured, photographed and pathologically examined.

### Bioinformatics Method

Putative target genes of miR-148a were obtained from TargetScan and PicTar database. The main criterion for target recognition is base pairing between the seed region of miRNA and the 3’UTR of its target. Basing on this rationale, 5 candidate genes (ATP6AP2, CCK-BR, MEOX2, MITF, SNN) that scored high in both algorithms were selected for experimental verification and CCK-BR protein was proved down-regulated by miR-148a mimics.

### Cloning of 3’UTR and Mutant 3’UTRs of CCK-BR into pMIR-REPORT Luciferase Vector

CCK-BR was predicted as a potential target gene of miR-148a using the TargetScan and PicTar algorithms. The 3’UTR of CCK-BR was amplified by PCR from SGC-7901 cell genomic DNA using the following primers: 5’-CATGAGCTCGTAGAGGGGCCGTGGGGGTT-3’ and 5’-CATAAGCTT GGAAGGAGAGGGCAGGGCCA-3’. The PCR product was isolated from the gel and subsequently cloned into the SecI and HindIII sites of the pMIR-REPORT luciferase vector (Applied Biosystems) and named pMIR/CCK-BR. After sequencing, this construct was used as the template for four mutant CCK-BR 3’UTRs which were amplified by PCR. The four pairs of primers for their respective first-round PCR product were as follows: 5’-CATGAGCTCGTAGAGGGGCCGTGGGGGTT-3’ and 5’-GGAAGGGTGACTCCTTGTCATT-3’; 5’-GATTAATGCCTCAGTTTGTTTT-3’ and 5’-CATAAGCTTGGAAGGAGAGGGCAGGGCCA-3’; 5’-CCCAATCACCTCAGTAAATACC-3’ and 5’-CATAAGCTTGGAAGGAGAGGGCAGGGCCA-3’; 5’-GCTGTTCAGGAGTCAAAAGGTT-3’ and 5’-CATAAGCTTGGAAGGAGAGGGCAGGGCCA-3’; Then, the four first-round PCR products were used as forward or reverse primer matched respectively with following primers: 5’-CATAAGCTTGGAAGGAGAGGGCAGGGCCA-3’; 5’-CATGAGCTCGTAGAGGGGCCGTGGGGGTT-3’; 5’-CATGAGCTCGTAGAGGGGCCGTGGGGGTT-3’; 5’-CATGAGCTCGTAGAGGGGCCGTGGGGGTT-3’. The second-round PCR was performed with the four newly matched-pairs of primers described above. Four second-round PCR products were cloned into the SecI and HindIII sites of the pMIR-REPORT luciferase vector respectively and named pMIR/CCK-BR/mut1, pMIR/CCK-BR/mut2, pMIR/CCK-BR/mut3 and pMIR/CCK-BR/mut4. All constructs were verified by sequencing.

### Luciferase Activity Assay

As described in detail previously [18], luciferase activity was measured by using dual-luciferase reporter assay (Promega, Madison, WI, USA) according to the manufacturer’s instructions.

### Western Blot

Cells in culture and tissue samples were respectively lysed using M-PER reagent (Pierce, Rockford, IL, USA) or T-PER reagent (Pierce) in the presence of Cocktail protease inhibitor (Pierce). Western blot was carried out as previously described [16]. Rabbit polyclonal antibody against STAT3, p-STAT3 (Tyr705), Akt, p-Akt (Ser473) or CCK-BR (1:1000, Abcam, Cambridge, UK), HRP-conjugated secondary antibody (1:5000, Santa Cruz Biotechnology, Santa Cruz, CA, USA) were used. GAPDH (1:5000, Kangchen, Shanghai, China) was simultaneously used as an internal control. Signals were detected and visualized with Immobilon Western chemiluminescent HRP Substrate (Millipore, Bedford, MA, USA).

### Statistical Analysis

The relationship between the miR-148a expression level and clinicopathologic parameters was explored by the Pearson *x*^2^ test. The differences between groups were analyzed using Student *t* test, when there were only two groups, or assessed by one-way ANOVA when there were more than two groups. All statistical analyses were performed using the SPSS 15.0 software package. A two-tailed value of P < 0.05 was considered statistically significant.

## Results

### MiR-148a expression is Down-regulated in Gastric Cancer and Correlates with Clinicopathologic Parameters

**Fig 1:**
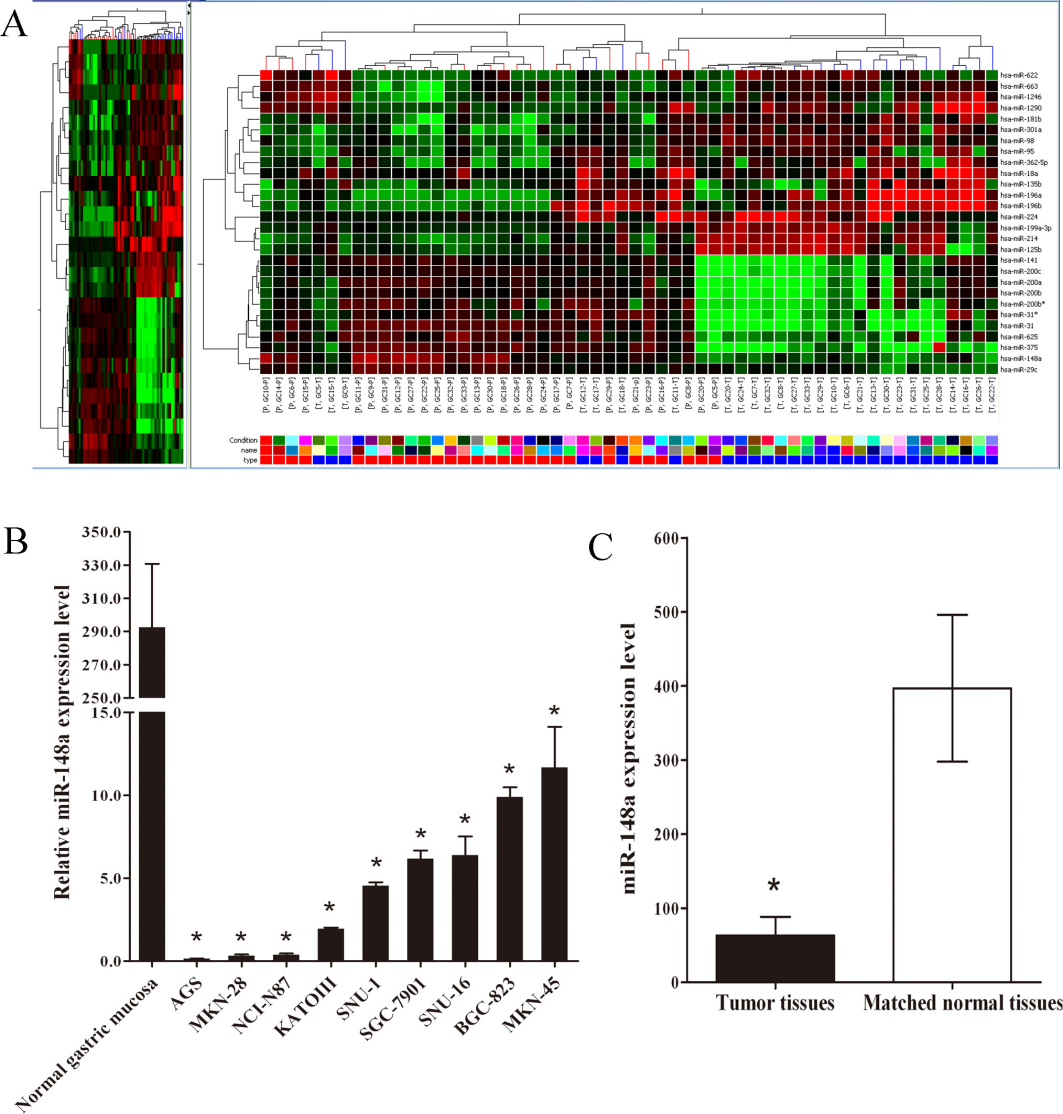
MiR-148a expression was down-regulated in gastric cancer tissues and gastric cancer cell lines compared with the corresponding controls. (A) MiRNA microarray assay was carried out using 28 surgical specimens of gastric cancer tissues. (B) QRT-PCR was carried out using nine gastric cancer cell lines and one pooled normal gastric mucosa tissue. The mean and standard deviation of miR-148a expression levels are shown. The data represent triplicate measurements from single RNA samples (*, P < 0.05, compared with pooled normal gastric mucosa). (C) QRT-PCR was carried out using 41 surgical specimens of gastric cancer tissues (black bar) and matched normal tissues (white bar). The mean and standard deviation of miR-148a expression levels are shown. The data represent triplicate measurements from single RNA samples (*, P < 0.05).

To explore the role of miRNAs in gastric cancer, we performed miRNA microarray profiling in 28 pairs of gastric cancer tissues and the matched normal mucosal tissues. We have submitted our dataset in the repository of “Gene Expression Omnibus” and the accession number was “GSE78775” (http://www.ncbi.nlm.nih.gov/geo/query/acc.cgi?acc=GSE78775). Cluster analysis based on the miRNA expression pattern indicated a significant difference between gastric cancer tissues and the matched normal mucosal tissues (Fig 1A). The miRNA microarray identified 17 miRNAs that were up-regulated in gastric cancer (relative expression ratio > 2.0) and 11 miRNAs that were down-regulated compared with their matched normal tissues (relative expression ratio < 0.5) (Table 1). It is known that epithelial-mesenchymal transition (EMT) has a critical role in metastasis which is responsible for early recurrence and poor survival of malignancies and cancer stem cells (CSCs) have the capacity to produce new tumors. For this reason, among the aberrantly expressed miRNAs, miR-148a was chosen as the candidate for further study for its suppressive effects to EMT and the CSCs-like properties in several types of human cancer [19–21]. In our previous study, we also found that miR-148a was down-regulated in gastric cancer cell lines relative to normal gastric mucosa [15]. All these strengthened our decision for choosing miR-148a for further research.

**Table 1:** Up- and down-regulated miRNAs in microarray.

up-regulated miRNAs			down-regulated miRNAs		
No.	miRNA	T/N fold change	No.	miRNA	T/N fold change
1	hsa-miR-196a	7.90	1	hsa-miR-375	0.12
2	hsa-miR-196b	4.59	2	hsa-miR-31	0.14
3	hsa-miR-214	3.30	3	hsa-miR-148a	0.31
4	hsa-miR-301a	2.76	4	hsa-miR-31*	0.34
5	hsa-miR-1290	2.74	5	hsa-miR-200a	0.38
6	hsa-miR-181b	2.67	6	hsa-miR-200c	0.39
7	hsa-miR-622	2.39	7	hsa-miR-141	0.40
8	hsa-miR-663	2.32	8	hsa-miR-625	0.41
9	hsa-miR-224	2.28	9	hsa-miR-200b*	0.41
10	hsa-miR-1246	2.25	10	hsa-miR-200b	0.46
11	hsa-miR-18a	2.22	11	hsa-miR-29c	0.48
12	hsa-miR-135b	2.18			
13	hsa-miR-95	2.12			
14	hsa-miR-98	2.10			
15	hsa-miR-125b	2.09			
16	hsa-miR-199a-3p	2.07			
17	hsa-miR-362-5p	2.06			

To validate the expression trend of miR-148a in the gastric cancer cell lines and gastric cancer tissues obtained from miRNA microarray assay, quantitative real-time RT-PCR (qRT-PCR) was performed to detect miR-148a in nine gastric cancer cell lines and a pooled normal gastric mucosa tissue. As shown in Fig 1B, miR-148a expression in these nine gastric cancer cell lines was significantly lower than that in a pooled normal gastric mucosa tissue. Furthermore, we detected miR-148 expression in 41 pairs of gastric cancer tissues and the matched normal mucosal tissues. As shown in Fig 1C, miR-148a was dramatically down-regulated in gastric cancer tissues compared with matched normal tissues (63.34 ± 25.23 *vs* 396.82 ± 98.98, P < 0.05). Collectively, these results provided sufficient evidence that miR-148a was prominently down-regulated in gastric cancer.

Extensive analysis showed that, of the 41 gastric cancer tissues, 73% (30/41) showed miR-148a down-regulation in comparison with the matched normal mucosal tissues (relative expression ratio < 1.0). Moreover, 54% (22/41) of the tumor tissues showed more significant down-regulation of miR-148a (relative expression ratio < 0.5). Basing on relative expression ratios of < 0.5, the 41 clinical cases were divided into two groups: miR-148a low expression group (n = 22) and miR-148a high expression group (n = 19). We found that the low miR-148a expression group frequently had more lymph node metastasis (P = 0.017, Table 2).

**Table 2:** Relationship between miR-148a expression level and clinicopathologic parameters in 41 gastric cancer patients.

Clinicopathological parameters	miR-148a expression		P*
	High (n = 19)	Low (n = 22)	
Age (years)			
≤60	8	12	0.427
>60	11	10	
Gender			
Male	14	18	0.803
Female	5	4	
Location			
Distal third	12	9	0.155
Middle third, proximal third	7	13	
Differentiation			
Well-differentiated, modetately	5	10	0.205
Poory differentiated	14	12	
Histologic type			
Intestinal	18	16	0.147
Diffuse	1	6	
Local invasion			
T1, T2	3	4	1.000
T3, T4	16	18	
Lymph node metastasis			
N0, N1	14	8	0.017
N2, N3	5	14	
TNM stage			
Ⅰ, Ⅱ	8	4	0.093
Ⅲ, Ⅳ	11	18	

miR-148a expression level associated with clinicopathological features, including tumor location, differentiation, histologic type, local invasion, lymph node metastasis and tumor-node-metastasis (TNM) stage was shown. Statistical significance was assessed by Pearson *x*^2^ test.

*, Pearson *x*^2^ test.

### MiR-148a Inhibits Proliferation and Migration of Gastric Cancer Cells

**Fig 2:**
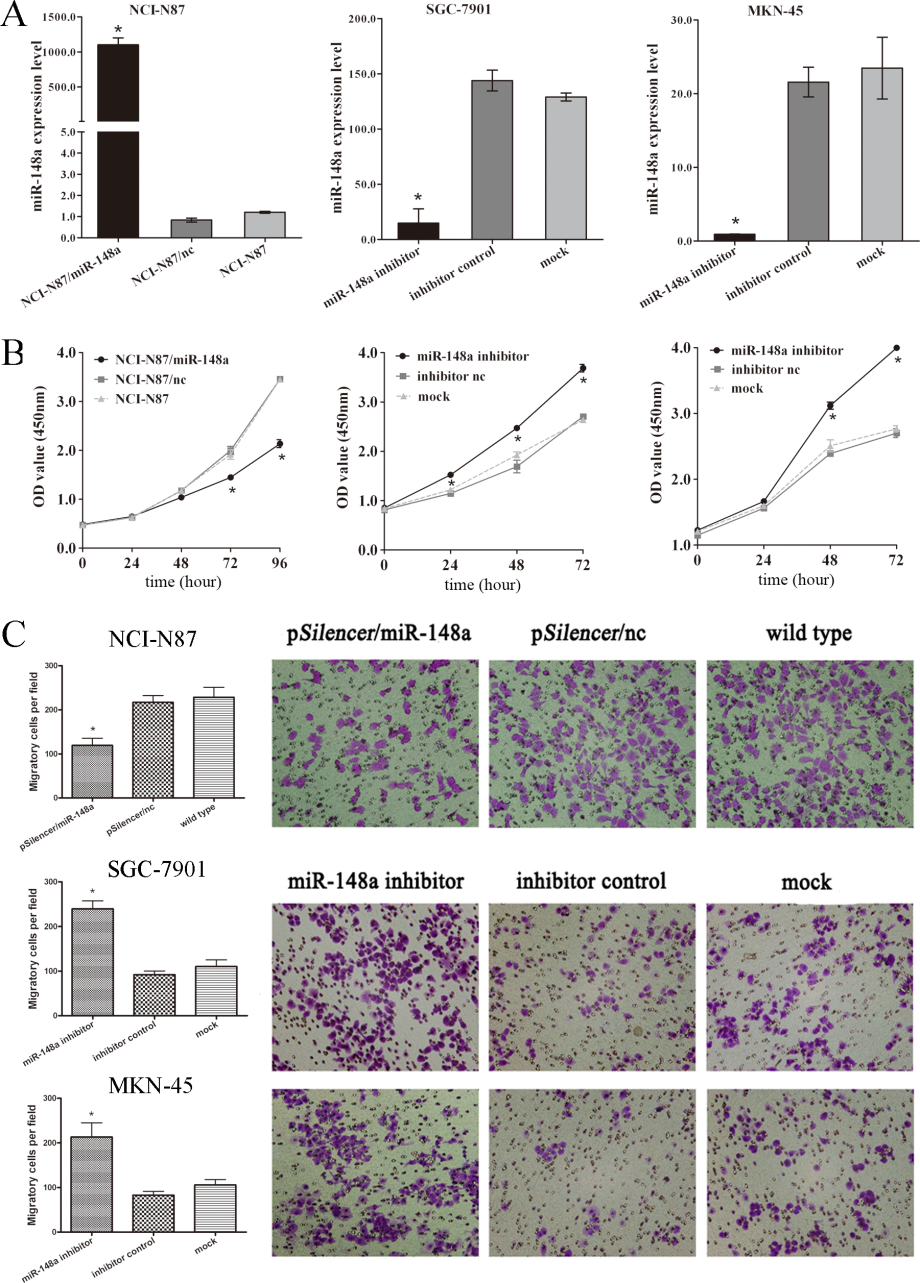
The effect of miR-148a on the proliferation and migration of NCI-N87, SGC-7901 and MKN-45 cells. (A) Transfection efficiency was assessed by qRT-PCR. The results showed that miR-148a expression vector (p*Silencer*/miR-148a) and miR-148a inhibitor could dramatically affect miR-148a expression in gastric cell lines. Results are means of three independent experiments ± S.D. (*, P < 0.05). (B) Cell proliferation was measured by CCK-8. NCI-N87 cells stably transfected with p*Silencer*/miR-148a grew more slowly than the control or wild type group; SGC-7901 and MKN-45 cells transfected with miR-148a inhibitors grew faster than their respective controls. Points, average of three independent experiments; bars, S.D. (*, P < 0.05). (C) Up-regulation of miR-148a depressed migration of NCI-N87 cells; SGC-7901 and MKN-45 cells migrated faster after miR148a knockdown. Average migratory cell number of three independent experiments ± S.D. (*, P < 0.05).

Given the down-regulation of miR-148a in gastric cancer tissues, we predicted that miR-148a may function as a tumor suppressor. To verify our hypothesis, ectopic expression as well as knockdown of miR-148a was carried out in gastric cancer cell lines. QRT-PCR was used to ensure the transfection efficiency (Fig 2A). NCI-N87 cells stably transfected with pSilencer/miR-148a grew more slowly than the control or the parental cells group (Fig 2B). Conversely, SGC-7901 and MKN-45 cells transfected with miR-148a inhibitor grew faster than the control and the parental cells, respectively (Fig 2B). These results indicated that miR-148a slower the proliferation in gastric cancer cells.

We further evaluated the effects of miR-148a on cell migration, which also plays a vital role in malignant progress and metastasis. As shown in Fig 2C, ectopic expression of miR-148a suppressed migration of NCI-N87 (pSilencer/miR-148a group, 119 ± 16 cells per field; pSilencer/nc group, 217 ± 16 cells per field; wild type group, 228 ± 23 cells per field; P < 0.05) and, conversely, miR-148a knockdown accelerated migration of SGC-7901 (miR-148a inhibitor group, 239 ± 18 cells per field; inhibitor control group, 92 ± 8 cells per field; mock group, 110 ± 15 cells per field; P < 0.05) and MKN-45 (miR-148a inhibitor group, 213 ± 32 cells per field; inhibitor control group, 82 ± 9 cells per field; mock group, 105 ± 12 cells per field; P < 0.05). From these results, we concluded that miR-148a functions as a suppressor in cell proliferation and migration in gastric cancer cells.

### Ectopic Expression of miR-148a Inhibits Tumorigenicity *in vivo*

**Fig 3:**
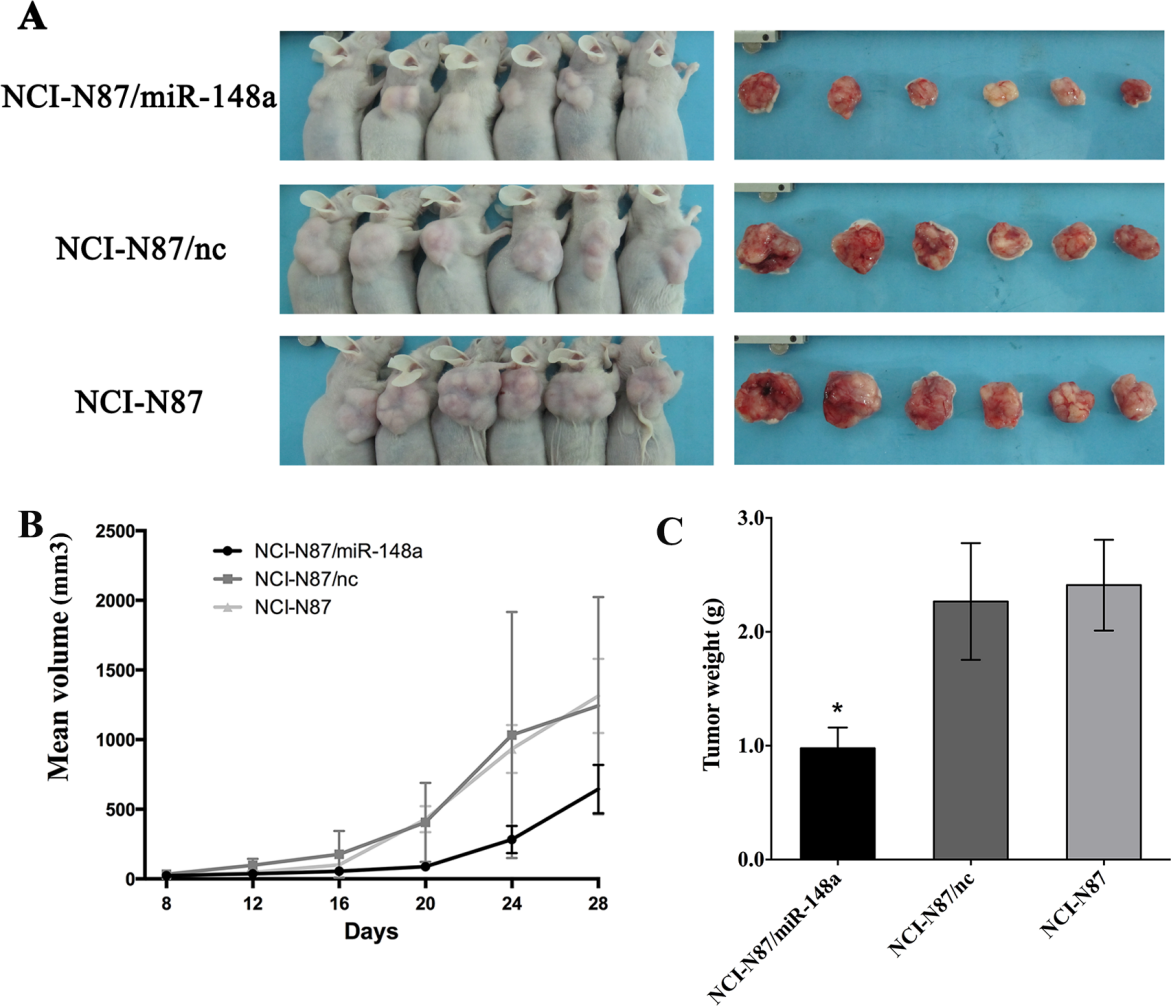
MiR-148a inhibited tumor growth *in vivo*. (A) Photographs of tumors derived from NCI-N87/miR-148a (NCI-N87 cells stably transfected with p*Silencer*/miR-148a vector), NCI-N87/nc (NCI-N87 cells stably transfected with p*Silencer*/nc vector) or wild type (parent NCI-N87 cells) in nude mice. (B) Tumor growth curves showed that tumors derived from NCI-N87/miR-148a cells (black line) in nude mice grew more slowly than the control group (dark grey line) or the parental cells (light grey line). Bars, S.D. (C) Average weight of tumors derived from NCI-N87/miR-148a, NCI-N87/nc or wild type NCI-N87 cells. Means ± S.D. are shown (*, P < 0.05).

We next examined whether enforced miR-148a expression could suppress tumor growth *in vivo*. NCI-N87/miR-148a, NCI-N87/nc or NCI-N87 cells were injected into male nude mice, and tumor formation was monitored. After 4 weeks, the mice were sacrificed and the tumors were weighted. The results showed that the tumor formation was significantly inhibited in mice injected with NCI-N87/miR-148a cells in comparison with the control or the parental cells (Fig 3A and 3B and S1 Table). The average tumor weight in NCI-N87/miR-148a group was 978.3 ± 181.3 mg, which was significantly lower (P < 0.05) than that in NCI-N87/nc group (2267.0 ± 512.5 mg) or that in NCI-N87 group (2410.0 ± 398.3 mg; Fig 3C and S2 Table). Thus, our data demonstrated that ectopic expression of miR-148a suppresses tumorigenicity *in vivo*.

### MiR-148a Targets CCK-BR Directly

**Fig 4:**
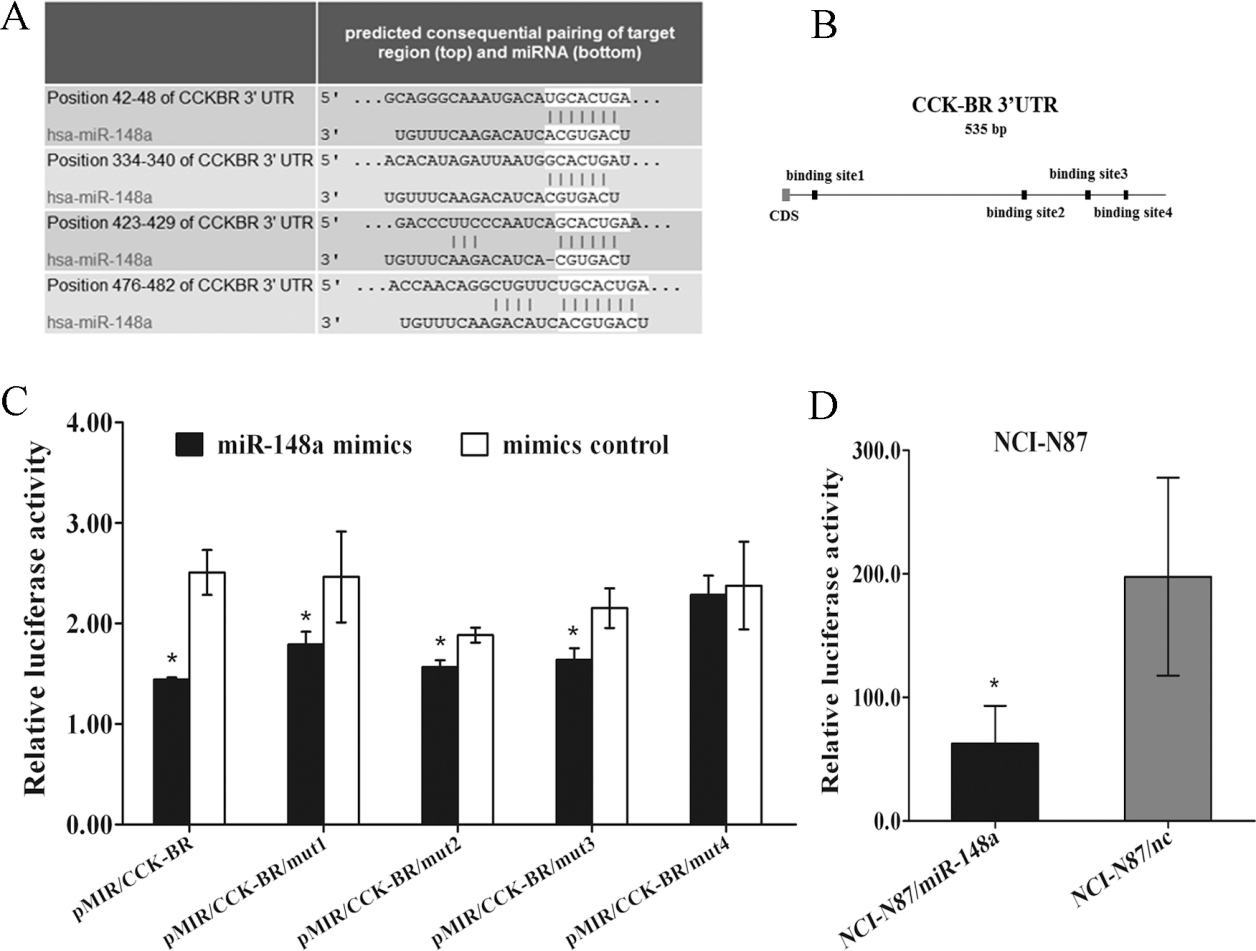
CCK-BR is a validated target of miR-148a. (A, B) Putative binding sites of miR-148a in the CCK-BR 3’UTR (white sequences) predicted by TargetScan. (C) MiR-148a mimics depressed the relative luciferase activity control by wild-type CCK-BR 3’UTR, which could be eliminated by nucleotide mutations in BS4 but not BS1, BS2 or BS3 sequences. Means ± S.D. are shown (*, P < 0.05). (D) The relative luciferase activity in NCI-N87 stably transfected with p*Silencer*/miR-148a was lower than that in NCI-N87 stably transfected with p*Silencer*/nc. Means ± S.D. are shown. (*, P < 0.05).

To explore the molecular mechanism by which miR-148a functions in gastric carcinogenesis, TargetScan and PicTar algorithms were used and 5 predicted target genes (ATP6AP2, CCK-BR, MEOX2, MITF, SNN) attracted our attention for their high scores in both algorithms (S3 and S4 Tables). Western blot was carried out and CCK-BR protein was significantly down-regulated by miR-148a mimics. Moreover, previous research proved that the gastrin and CCK-BR loop blockage inhibited gastric cancer cells proliferation [22]. Thus, CCK-BR was selected for further investigation. CCK-BR, a receptor mainly for Cholecystokinin-B (CCK-B) and gastrin, that can functionally couple with intracellular signaling molecules [23], was evaluated and four predicted miR-148a binding sites (named BS1, BS2, BS3 and BS4 respectively) reside within 3’UTR (Fig 4A and 4B). Therefore, we further tested whether CCK-BR was a direct target of miR-148a in gastric cancer.

To experimentally validate whether CCK-BR was a direct target of miR-148a, a wide-type 3’UTR fragment of CCK-BR was cloned downstream of *Firefly* luciferase gene in the pMIR-REPORT luciferase vector. Since four predicted miR-148a binding sites reside within CCK-BR 3’UTR, four mutant 3’UTR fragments were engineered and cloned downstream of Firefly luciferase gene and the four resulting constructs were pMIR/CCK-BR/mut1, pMIR/CCK-BR/mut2, pMIR/CCK-BR/mut3 or pMIR/CCK-BR/mut4. CCK-BR 3’UTR reporter construct and four mutant 3’UTR reporter constructs were co-transfected into HEK 293T cells with miR-148a mimics and the luciferase activity was detected on 48h post-transfection. Dual-luciferase reporter assays revealed that enforced miR-148a expression significantly attenuated the activity of firefly luciferase with the wild-type 3’UTR, mutant BS1 3’UTR, mutant BS2 3’UTR and BS3 mutant 3’UTR, whereas this effect was abrogated when the BS4 sequence was mutated (Fig 4C). Subsequent studies disclosed that the luciferase activity of NCI-N87/miR-148a cells with enforced miR-148a expression was significantly attenuated (Fig 4D). Taken together, these data suggest that miR-148a may inhibit the expression of CCK-BR by directly binding to its BS4 site within 3’UTR.

### MiR-148a Expression Correlates inversely with CCK-BR Protein Expression in Gastric Cancer

**Fig 5:**
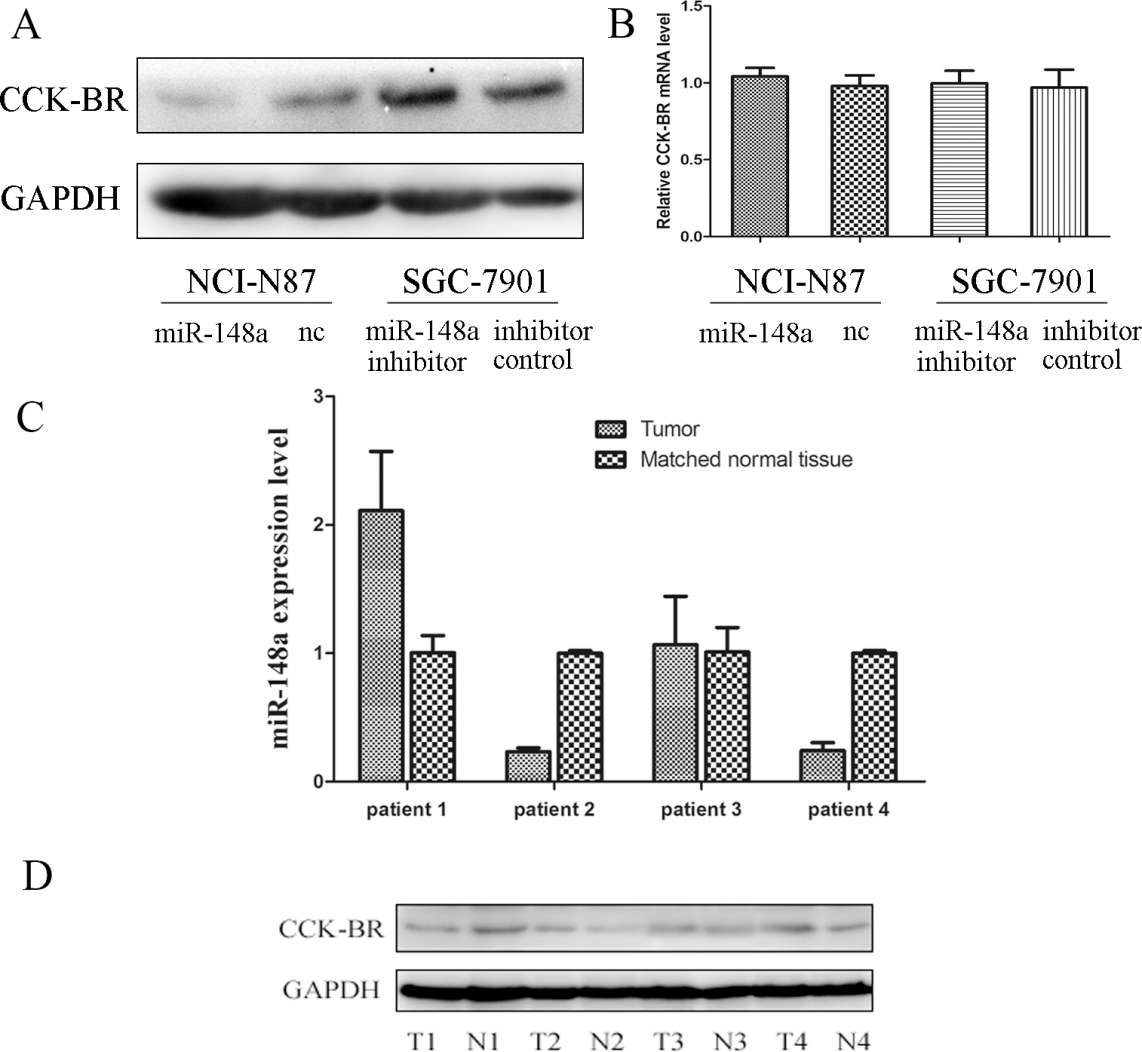
MiR-148a expression correlates inversely with CCK-BR protein expression in gastric cancer. (A) CCK-BR protein was detected in NCI-N87 cells stably transfected by p*Silencer*/miR-148a (or p*Sliencer*/nc vector) or in SGC-7901 cells transfected by miR-148a inhibitor (or inhibitor control). GAPDH was used as an internal loading control. (B) CCK-BR mRNA was detected in NCI-N87 cells stably transfected by p*Silencer*/miR-148a or p*Sliencer*/nc vector and SGC-7901 cells transfected by miR-148a inhibitor or inhibitor control. The results are shown as fold changes relative to the control. The data represent triplicate measurements from single RNA samples. (C) QRT-PCR analysis of CCK-BR in four paired tumor/matched normal tissues. The data represent triplicate measurements from single RNA samples. (D) Western blot analysis of CCK-BR in four paired tumor/normal tissues (T/N).

It is known that miRNAs can regulate gene expression through decreased translation of target mRNA, increased degradation of target mRNA, or both. To confirm whether CCK-BR protein expression could be negatively regulated by miR-148a, western blot and qRT-PCR assays were performed. As shown in Fig 5A, CCK-BR protein level was reduced in NCI-N87/miR-148a cells in comparison with the control group. Conversely, CCK-BR protein level in SGC-7901 cells was up-regulated by miR-148a inhibitor transfection. Nevertheless, qRT-PCR analysis revealed that the CCK-BR mRNA level was not decreased or increased by miR-148a compared with the control (Fig 5B). These data provided strong evidence to support that miR-148a negatively regulates CCK-BR expression at the post-transcription level.

In addition, we measured miR-148a expression level and CCK-BR protein level in 4 pairs of gastric cancer tissues and the matched normal mucosal tissues. As shown in Fig 5C and 5D, an inverse correlation was observed between CCK-BR expression and the miR-148a expression level.

### Knockdown of CCK-BR Inhibits Proliferation and Migration of Gastric Cancer Cells

**Fig 6:**
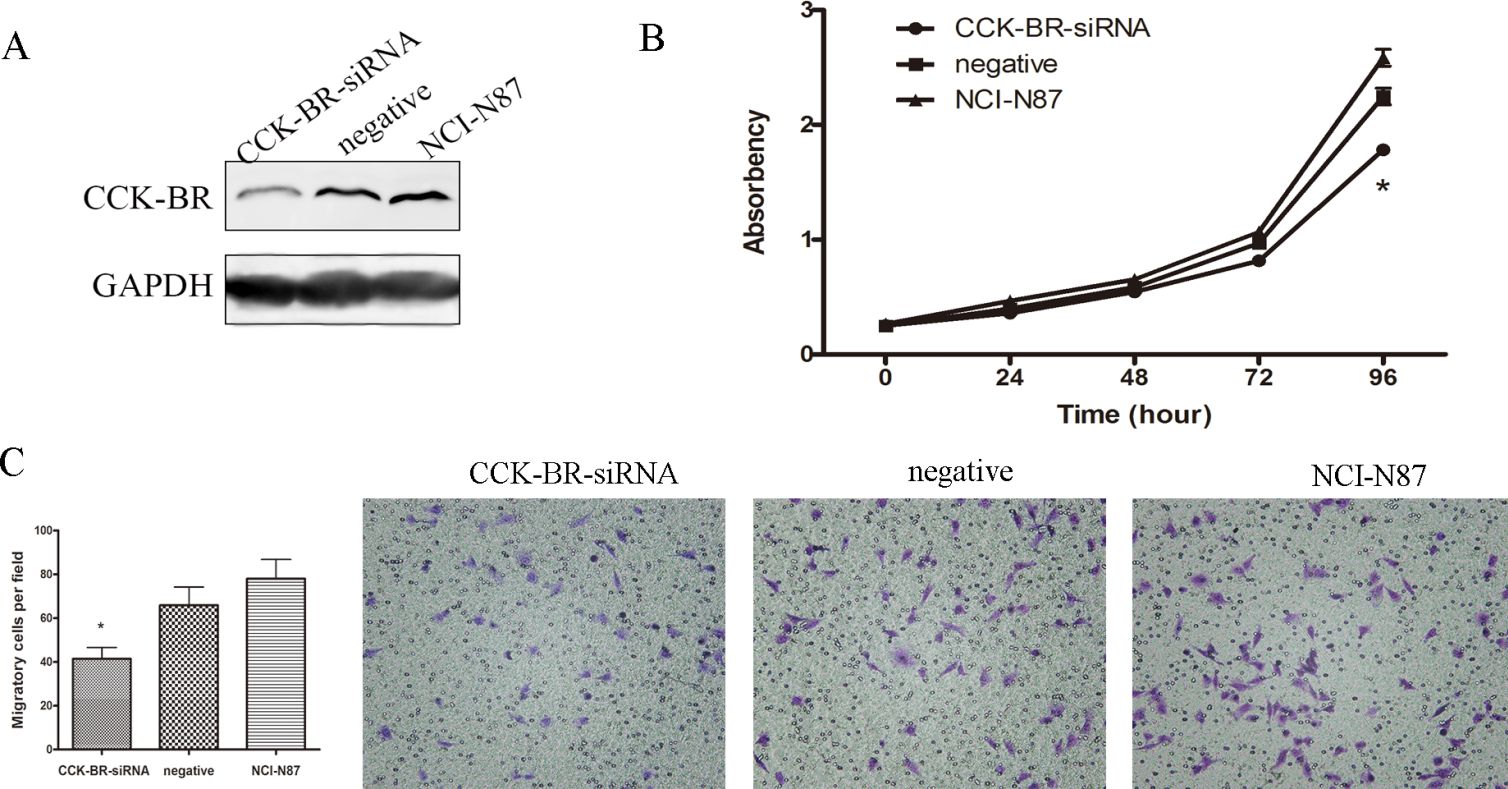
The effect of miR-148a can be rescued by down-expressed CCK-BR. (A) Western blot was used to monitor the protein level of CCK-BR in NCI-N87 cells 48 h after transfection with CCK-BR siRNA (or negative control). (B) Effects of CCK-BR knockdown on cell proliferation in NCI-N87 cells. Means ± S.D. are shown (*, P < 0.05). (C) Migration assay in NCI-N87 cells with CCK-BR siRNA or negative control. Means ± S.D. are shown (*, P < 0.05).

Since CCK-BR was verified as the target gene of miR-148a in gastric cancer, specific knockdown of CCK-BR should elicit similar phenotypes induced by miR-148a in gastric cancer cells. Therefore, CCK-BR knockdown experiment was performed to determine whether knockdown of CCK-BR has its negative regulatory function on proliferation and migration in gastric cancer cells. For this purpose, NCI-N87 cells were transfected with siRNA, and as a result, CCK-BR protein expression was diminished in NCI-N87 cells (Fig 6A). As expected, proliferation assay showed that CCK-BR knockdown significantly inhibited cell proliferation (Fig 6B). Similarly, cell migration was significantly reduced by CCK-BR knockdown (CCK-BR-siRNA group, 41 ± 5.0 cells per field; negative group, 66 ± 8.0 cells per field; NCI-N87 group, 78 ± 9.0 cells per field; P < 0.05, Fig 6C).

### MiR-148a Inhibits Activation of STAT3 and Akt in Gastric Cancer Cells

**Fig 7:**
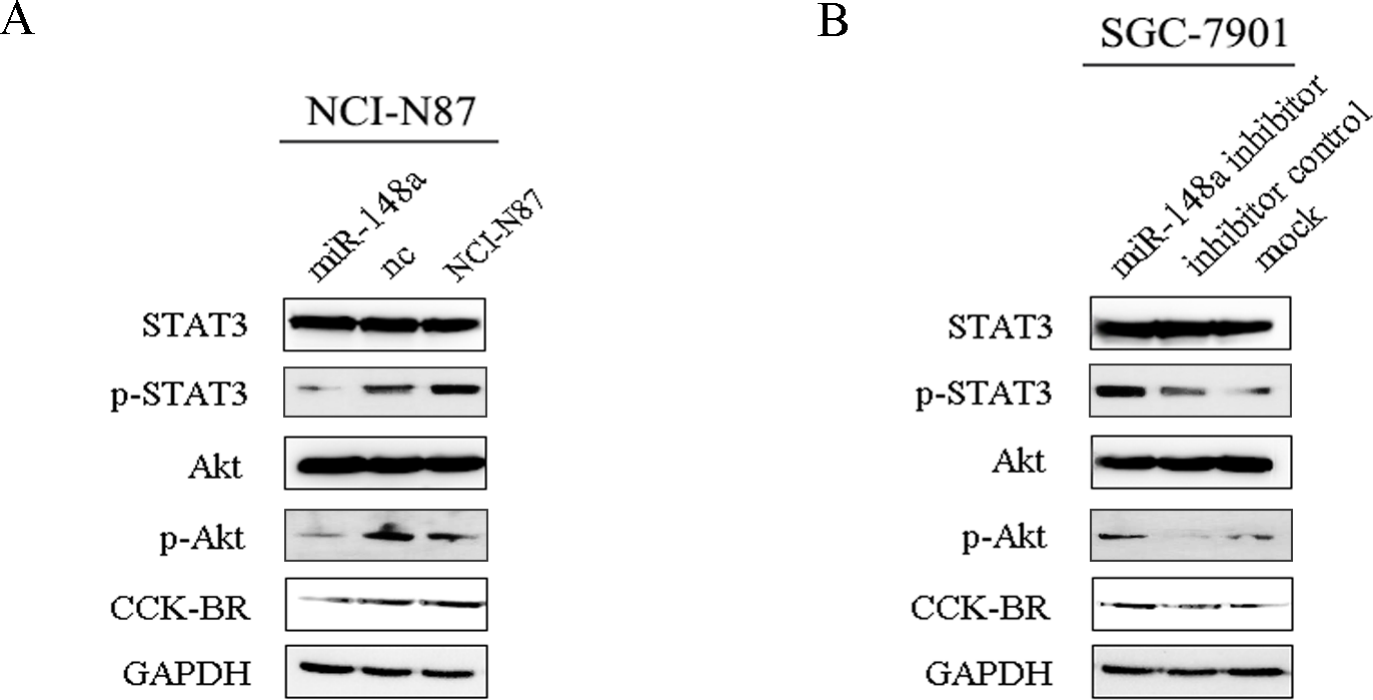
MiR-148a inhibits activation of STAT3 and Akt in gastric cancer cells. (A) Western blot indicated alterations of STAT3, p-STAT3, Akt, p-Akt, CCK-BR protein levels in NCI-N87/miR-148a, NCI-N87/nc and NCI-N87 group. (B) Western blot showed changes of STAT3, p-STAT3, Akt, p-Akt, CCK-BR protein levels in SGC-7901/miR-148a inhibitor, NCI-N87/inhibitor control and mock group.

Given the important role of STAT3 and Akt activation in cell growth, proliferation and survival in many human cancers [24–27], including gastric cancer [28], further studies were designed to explore the effects of miR-148a on activation of STAT3 and Akt in gastric cancer cells. Western blot was performed to detect the protein levels of STAT3, phosphorylated-STAT3 (p-STAT3), Akt, phosphorylated-Akt (p-Akt) and CCK-BR in gastric cancer cells. We confirmed that STAT3 and Akt protein expression were constitutively active. However, enforced miR-148a expression effectively down-regulated p-STAT3 and p-Akt protein levels in NCI-N87/miR-148a cells (Fig 7A). Conversely, down-regulation of miR-148a significantly enhanced p-STAT3 and p-Akt protein levels in SGC-7901 cells (Fig 7B). Further, CCK-BR, STAT3, p-STAT3, Akt and p-Akt expression level were detected in the tumor sections removed from the mice. As S1 Fig shown, CCK-BR, p-STAT3 and p-Akt protein levels were decreased in the tumor tissues in the NCI-N87/miR-148a group.

Taken together, our results suggest that miR-148a may inhibit proliferation and migration by targeting CCK-BR via inactivating STAT3 and Akt, as well as subsequent modulation of their downstream molecules.

## Discussion

Previous research has shown that miRNAs play a crucial role in several types of human cancer. Using miRNA microarray, we identified the miRNA expression profile in gastric cancer tissues in comparison with the matched normal mucosal tissues, and miR-148a was found significantly down-regulated in cancer tissues. Our data imply its potential role in gastric carcinogenesis. In this study, we aimed to clarify the function and molecular mechanism of miR-148a in the initiation and progress of gastric cancer.

Low expression of miR-148a in gastric cancer tissues indicated that miR-148a may function as a tumor suppressor. Therefore, we predicted that ectopic expression of miR-148a could suppress the oncogenic activity of gastric cancer cell lines. The *in vitro* and *in vivo* assay results confirmed that miR-148a could inhibit proliferation, migration in gastric cancer cells and tumor formation in nude mice, respectively. Similar results have been obtained in several other types of human malignant disease. The silencing of miR-148a expression by DNA hypermethylation was crucial in early pancreatic carcinogenesis, indicating miR-148a’s role as a tumor suppressor [29]. MiR-148a involves in DNA methylation process via targeting DNMT-1 and, more importantly, serves as a tumor suppressor in hepatocellular carcinogenesis [13, 30–32].

MiRNAs post-transcriptionally regulate gene expression by inhibition of protein synthesis or/and degradation of target mRNA. To explore the mechanism responsible for proliferation and migration-suppressive effects of miR-148a, TargetScan and PicTar database were used and CCK-BR was identified as the potential target of miR-148a. CCK-BR is prominently expressed in gastric mucosa and brain and involved in some human malignant diseases [33–35]. Song *et al* reported that miR-148b can suppress cell growth by targeting CCK-BR in colorectal cancer [36]. In our study, CCK-BR was validated as a target gene of miR-148a. Up-regulation of miR-148a depressed CCK-BR protein level in gastric cancer cells, and down-regulation of miR-148a enhanced CCK-BR protein level. In addition, we detected reduced luciferase activity from wild-type 3’UTR construct in HEK 293T cells, however, this effect could be diminished when BS4 sequence was mutated. Moreover, we disclosed that CCK-BR protein level in gastric cancer tissues was significantly higher than in the matched normal mucosal tissues and, consistently, miR-148a expression level in gastric tumor tissues was lower. Further, knockdown of CCK-BR can mimic proliferation and migration -suppressive effects induced by enhanced miR-148a expression. Taken together, these data indicated that miR-148a inhibits proliferation, migration and tumorigenicity in gastric cancer cells via targeting CCK-BR.

Recently, many new insights into the core signaling pathways in gastric cancer have been made, including STAT3 and Akt pathways [37–40]. These pathways are often constitutively activated in subsets of human gastric cancer tissues and cell lines. Previous study has reported that gastrin acting on CCK-BR induces cyclooxygenase-2 expression through JAK2/STAT3/PI3K/Akt pathway in human gastric cancer cells [28]. The Chen group demonstrated that JNK phosphorylates STAT3, which, in turn, activates Akt and induces VEGF expression and cell migration, and they validated the JNK regulation of STAT3 via miR-21 and link the JNK-STAT3-Akt signaling axis to the phosphorylation of EZH2 [41, 42]. In our study, we disclosed that miR-148a inhibits cell proliferation and migration by targeting CCK-BR through inactivation of STAT3 and/or Akt pathway, resulting in inactivation of multiple downstream survival factors.

Some current studies have revealed the miR-148a-involved mechanism underlying human gastric carcinogenesis with ROCK1, DNMT1 and MMP7 as the direct targets of miR-148a [32, 43, 44]. Although these studies obtained similar results to ours, our work not only reinforces miR-148a’s role as a tumor suppressor in human gastric carcinogenesis, but also unveils a new miR-148a-involved mechanism in gastric carcinogenesis for the first time. Our job clarified that miR-148a is down-regulated in human gastric cancer and functions as a suppressor in cell proliferation, migration *in vitro* and tumor formation *in vivo*. More importantly, we successfully proved that miR-148a directly binds CCK-BR and conduct its anti-cancer effects via inactivating STAT3 and Akt, as well as subsequent modulation of their downstream gene products. Deciphering the molecular basis of miR-148a’s role in human gastric cancer may extend our understanding in molecular mechanism underlying gastric carcinogenesis, and lay a theoretical foundation for further exploration in early diagnosis, clinical behavior prediction, chemotherapy and biotherapy.

## Supporting Information

S1 Fig MiR-148a inhibits activation of STAT3 and Akt in xenograft tumor tissues. Western blot analysis of STAT3, p-STAT3, Akt, p-Akt and CCK-BR protein levels in the xenograft tumor tissues of NCI-N87/miR-148a, NCI-N87/nc and NCI-N87 groups.
(TIF)

S2 Fig The uncropped blots from which the figure panels were made. (TIF)

S1 Table Tumor volume of mice in NCI-N87, NCI-N87/nc and NCI-N87/miR-148a groups. Measured at day 8, 12, 16, 20, 24 and 28 after injection.
(XLSX)

S2 Table Tumor weight of mice in NCI-N87, NCI-N87/nc and NCI-N87/miR-148a groups. (XLSX)

S3 Table Putative target genes list from TargetScan database. ATP6AP2, CCKBR, MEOX2, GLRX5, TNFRSF6B, C5orf30, SNN, GADD45A, ARL8B, PDE1C, NPTN, NPEPL1, MITF, CLCN6, KRT76, CDK5R1, AK2, TMEM54, C18orf25, SOS2 … …
(XLSX)

S4 Table Putative target genes list from PicTar database. CCBKR, SFRS2IP, MITF, RPS6KA5, ATP6AP2, CGI-109, KIS, DKFZp566C0424, MEOX2, SYNJ1, INO80, TNRC6, SDFR1, ROBO1, PLAA, SNN, USP6, ARL10C, ELF5, MTF1 … …
(XLSX)

## References

[1] Bickenbach K, Strong VE. Comparisons of Gastric Cancer Treatments: East vs. West. Journal of gastric cancer. 2012;12(2):55–62. 10.5230/jgc.2012.12.2.55 22792517 PMC3392325

[2] Bartel DP. MicroRNAs: target recognition and regulatory functions. Cell. 2009;136(2):215–33. 10.1016/j.cell.2009.01.002 19167326 PMC3794896

[3] Mohr AM, Mott JL. Overview of microRNA biology. Seminars in liver disease. 2015;35(1):3–11. 10.1055/s-0034-1397344 . 25632930 PMC4797991

[4] Borkowski R, Du L, Zhao Z, McMillan E, Kosti A, Yang CR, et al Genetic mutation of p53 and suppression of the miR-17 approximately 92 cluster are synthetic lethal in non-small cell lung cancer due to upregulation of vitamin D Signaling. Cancer research. 2015;75(4):666–75. 10.1158/0008-5472.CAN-14-1329 25519225 PMC4333022

[5] Zheng Q, Peskoe SB, Ribas J, Rafiqi F, Kudrolli T, Meeker AK, et al Investigation of miR-21, miR-141, and miR-221 expression levels in prostate adenocarcinoma for associated risk of recurrence after radical prostatectomy. The Prostate. 2014;74(16):1655–62. 10.1002/pros.22883 25252191 PMC4205269

[6] Hamada S, Masamune A, Miura S, Satoh K, Shimosegawa T. MiR-365 induces gemcitabine resistance in pancreatic cancer cells by targeting the adaptor protein SHC1 and pro-apoptotic regulator BAX. Cellular signalling. 2014;26(2):179–85. 10.1016/j.cellsig.2013.11.003 . 24216611

[7] Lin L, Liang H, Wang Y, Yin X, Hu Y, Huang J, et al microRNA-141 inhibits cell proliferation and invasion and promotes apoptosis by targeting hepatocyte nuclear factor-3beta in hepatocellular carcinoma cells. BMC cancer. 2014;14:879 10.1186/1471-2407-14-879 25425543 PMC4289273

[8] Hall DP, Cost NG, Hegde S, Kellner E, Mikhaylova O, Stratton Y, et al TRPM3 and miR-204 establish a regulatory circuit that controls oncogenic autophagy in clear cell renal cell carcinoma. Cancer cell. 2014;26(5):738–53. 10.1016/j.ccell.2014.09.015 25517751 PMC4269832

[9] Liu R, Gu J, Jiang P, Zheng Y, Liu X, Jiang X, et al DNMT1-microRNA126 epigenetic circuit contributes to esophageal squamous cell carcinoma growth via ADAM9-EGFR-AKT signaling. Clinical cancer research: an official journal of the American Association for Cancer Research. 2015;21(4):854–63. 10.1158/1078-0432.CCR-14-1740 . 25512445

[10] Visone R, Rassenti LZ, Veronese A, Taccioli C, Costinean S, Aguda BD, et al Karyotype-specific microRNA signature in chronic lymphocytic leukemia. Blood. 2009;114(18):3872–9. 10.1182/blood-2009-06-229211 19717645 PMC2773482

[11] Pan L, Huang S, He R, Rong M, Dang Y, Chen G. Decreased expression and clinical significance of miR-148a in hepatocellular carcinoma tissues. European journal of medical research. 2014;19(1):68 10.1186/s40001-014-0068-2 25444499 PMC4258268

[12] Tao S, He H, Chen Q, Yue W. GPER mediated estradiol reduces miR-148a to promote HLA-G expression in breast cancer. Biochemical and biophysical research communications. 2014;451(1):74–8. 10.1016/j.bbrc.2014.07.073 . 25063027

[13] Braconi C, Huang N, Patel T. MicroRNA-dependent regulation of DNA methyltransferase-1 and tumor suppressor gene expression by interleukin-6 in human malignant cholangiocytes. Hepatology. 2010;51(3):881–90. 10.1002/hep.23381 20146264 PMC3902044

[14] Xia J, Guo X, Yan J, Deng K. The role of miR-148a in gastric cancer. Journal of cancer research and clinical oncology. 2014;140(9):1451–6. 10.1007/s00432-014-1649-8 . 24659367 PMC11823805

[15] Yu BQ, Su LP, Li JF, Cai Q, Yan M, Chen XH, et al microrna expression signature of gastric cancer cells relative to normal gastric mucosa. Molecular medicine reports. 2012;6(4):821–6. 10.3892/mmr.2012.1006 . 22842726

[16] Feng R, Chen X, Yu Y, Su L, Yu B, Li J, et al miR-126 functions as a tumour suppressor in human gastric cancer. Cancer letters. 2010;298(1):50–63. 10.1016/j.canlet.2010.06.004 . 20619534

[17] Duan Y, Hu L, Liu B, Yu B, Li J, Yan M, et al Tumor suppressor miR-24 restrains gastric cancer progression by downregulating RegIV. Molecular cancer. 2014;13:127 10.1186/1476-4598-13-127 24886316 PMC4041902

[18] Zhang BG, Li JF, Yu BQ, Zhu ZG, Liu BY, Yan M. microRNA-21 promotes tumor proliferation and invasion in gastric cancer by targeting PTEN. Oncology reports. 2012;27(4):1019–26. 10.3892/or.2012.1645 22267008 PMC3583594

[19] Zhang JP, Zeng C, Xu L, Gong J, Fang JH, Zhuang SM. MicroRNA-148a suppresses the epithelial-mesenchymal transition and metastasis of hepatoma cells by targeting Met/Snail signaling. Oncogene. 2014;33(31):4069–76. 10.1038/onc.2013.369 . 24013226

[20] Wang SH, Li X, Zhou LS, Cao ZW, Shi C, Zhou CZ, et al microRNA-148a suppresses human gastric cancer cell metastasis by reversing epithelial-to-mesenchymal transition. Tumour biology: the journal of the International Society for Oncodevelopmental Biology and Medicine. 2013;34(6):3705–12. 10.1007/s13277-013-0954-1 . 23873106

[21] Jiang F, Mu J, Wang X, Ye X, Si L, Ning S, et al The repressive effect of miR-148a on TGF beta-SMADs signal pathway is involved in the glabridin-induced inhibition of the cancer stem cells-like properties in hepatocellular carcinoma cells. PloS one. 2014;9(5):e96698 10.1371/journal.pone.0096698 24806207 PMC4013140

[22] Zhou JJ, Chen ML, Zhang QZ, Zao Y, Xie Y. Blocking gastrin and CCK-B autocrine loop affects cell proliferation and apoptosis in vitro. Molecular and cellular biochemistry. 2010;343(1–2):133–41. 10.1007/s11010-010-0507-5 . 20559691

[23] Ito M, Matsui T, Taniguchi T, Tsukamoto T, Murayama T, Arima N, et al Functional characterization of a human brain cholecystokinin-B receptor. A trophic effect of cholecystokinin and gastrin. The Journal of biological chemistry. 1993;268(24):18300–5. . 8349705

[24] Corcoran RB, Contino G, Deshpande V, Tzatsos A, Conrad C, Benes CH, et al STAT3 plays a critical role in KRAS-induced pancreatic tumorigenesis. Cancer research. 2011;71(14):5020–9. 10.1158/0008-5472.CAN-11-0908 21586612 PMC3693754

[25] Lin L, Hutzen B, Zuo M, Ball S, Deangelis S, Foust E, et al Novel STAT3 phosphorylation inhibitors exhibit potent growth-suppressive activity in pancreatic and breast cancer cells. Cancer research. 2010;70(6):2445–54. 10.1158/0008-5472.CAN-09-2468 20215512 PMC2843552

[26] Ahmad A, Biersack B, Li Y, Kong D, Bao B, Schobert R, et al Deregulation of PI3K/Akt/mTOR signaling pathways by isoflavones and its implication in cancer treatment. Anti-cancer agents in medicinal chemistry. 2013;13(7):1014–24. . 23272911 10.2174/18715206113139990117

[27] Rojanasakul Y. Linking JNK-STAT3-Akt signaling axis to EZH2 phosphorylation: a novel pathway of carcinogenesis. Cell cycle. 2013;12(2):202–3. 10.4161/cc.23419 23287465 PMC3575446

[28] Xu W, Chen GS, Shao Y, Li XL, Xu HC, Zhang H, et al Gastrin acting on the cholecystokinin2 receptor induces cyclooxygenase-2 expression through JAK2/STAT3/PI3K/Akt pathway in human gastric cancer cells. Cancer letters. 2013;332(1):11–8. 10.1016/j.canlet.2012.12.030 . 23376640

[29] Hanoun N, Delpu Y, Suriawinata AA, Bournet B, Bureau C, Selves J, et al The silencing of microRNA 148a production by DNA hypermethylation is an early event in pancreatic carcinogenesis. Clinical chemistry. 2010;56(7):1107–18. 10.1373/clinchem.2010.144709 . 20431052

[30] Pan W, Zhu S, Yuan M, Cui H, Wang L, Luo X, et al MicroRNA-21 and microRNA-148a contribute to DNA hypomethylation in lupus CD4+ T cells by directly and indirectly targeting DNA methyltransferase 1. Journal of immunology. 2010;184(12):6773–81. 10.4049/jimmunol.0904060 . 20483747

[31] Long XR, He Y, Huang C, Li J. MicroRNA-148a is silenced by hypermethylation and interacts with DNA methyltransferase 1 in hepatocellular carcinogenesis. International journal of oncology. 2014;44(6):1915–22. 10.3892/ijo.2014.2373 . 24714841

[32] Yan J, Guo X, Xia J, Shan T, Gu C, Liang Z, et al MiR-148a regulates MEG3 in gastric cancer by targeting DNA methyltransferase 1. Medical oncology. 2014;31(3):879 10.1007/s12032-014-0879-6 . 24515776

[33] Sethi T, Herget T, Wu SV, Walsh JH, Rozengurt E. CCKA and CCKB receptors are expressed in small cell lung cancer lines and mediate Ca2+ mobilization and clonal growth. Cancer research. 1993;53(21):5208–13. . 8221657

[34] Jin G, Westphalen CB, Hayakawa Y, Worthley DL, Asfaha S, Yang X, et al Progastrin stimulates colonic cell proliferation via CCK2R- and beta-arrestin-dependent suppression of BMP2. Gastroenterology. 2013;145(4):820–30 e10. 10.1053/j.gastro.2013.07.034 23891976 PMC3829714

[35] Smith JP, Harms JF, Matters GL, McGovern CO, Ruggiero FM, Liao J, et al A single nucleotide polymorphism of the cholecystokinin-B receptor predicts risk for pancreatic cancer. Cancer biology & therapy. 2012;13(3):164–74. 10.4161/cbt.13.3.18698 22277584 PMC3336072

[36] Song Y, Xu Y, Wang Z, Chen Y, Yue Z, Gao P, et al MicroRNA-148b suppresses cell growth by targeting cholecystokinin-2 receptor in colorectal cancer. International journal of cancer Journal international du cancer. 2012;131(5):1042–51. 10.1002/ijc.26485 . 22020560

[37] Huang S, Chen M, Ding X, Zhang X, Zou X. Proton pump inhibitor selectively suppresses proliferation and restores the chemosensitivity of gastric cancer cells by inhibiting STAT3 signaling pathway. International immunopharmacology. 2013;17(3):585–92. 10.1016/j.intimp.2013.07.021 . 23973653

[38] Giraud AS, Menheniott TR, Judd LM. Targeting STAT3 in gastric cancer. Expert opinion on therapeutic targets. 2012;16(9):889–901. 10.1517/14728222.2012.709238 . 22834702

[39] Dun B, Sharma A, Teng Y, Liu H, Purohit S, Xu H, et al Mycophenolic acid inhibits migration and invasion of gastric cancer cells via multiple molecular pathways. PloS one. 2013;8(11):e81702 10.1371/journal.pone.0081702 24260584 PMC3829969

[40] Du W, Wang S, Zhou Q, Li X, Chu J, Chang Z, et al ADAMTS9 is a functional tumor suppressor through inhibiting AKT/mTOR pathway and associated with poor survival in gastric cancer. Oncogene. 2013;32(28):3319–28. 10.1038/onc.2012.359 . 22907434

[41] Chen F. JNK-induced apoptosis, compensatory growth, and cancer stem cells. Cancer research. 2012;72(2):379–86. 10.1158/0008-5472.CAN-11-1982 22253282 PMC3261582

[42] Chen B, Liu J, Chang Q, Beezhold K, Lu Y, Chen F. JNK and STAT3 signaling pathways converge on Akt-mediated phosphorylation of EZH2 in bronchial epithelial cells induced by arsenic. Cell cycle. 2013;12(1):112–21. 10.4161/cc.23030 23255093 PMC3570498

[43] Sakamoto N, Naito Y, Oue N, Sentani K, Uraoka N, Zarni Oo H, et al MicroRNA-148a is downregulated in gastric cancer, targets MMP7, and indicates tumor invasiveness and poor prognosis. Cancer science. 2014;105(2):236–43. 10.1111/cas.12330 24283384 PMC4317816

[44] Zheng B, Liang L, Wang C, Huang S, Cao X, Zha R, et al MicroRNA-148a suppresses tumor cell invasion and metastasis by downregulating ROCK1 in gastric cancer. Clinical cancer research: an official journal of the American Association for Cancer Research. 2011;17(24):7574–83. 10.1158/1078-0432.CCR-11-1714 . 21994419

