# Supplementary figures and images for: MiR-148a Functions as a Tumor Suppressor by Targeting CCK-BR via Inactivating STAT3 and Akt in Human Gastric Cancer
Source: PLoS One. 2016 Aug 12;11(8):e0158961. doi: 10.1371/journal.pone.0158961 (PMC4982598; doi:10.1371/journal.pone.0158961)

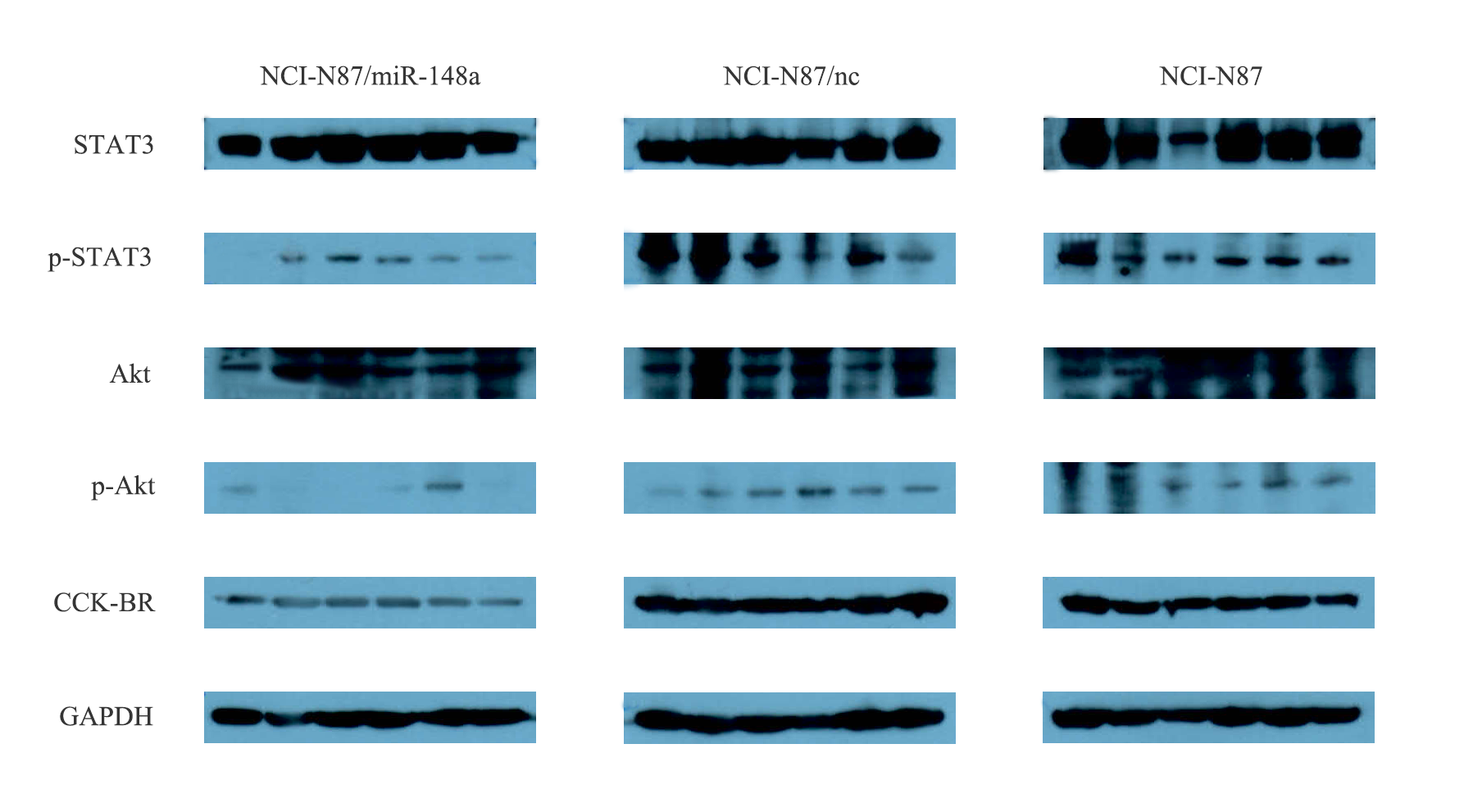

Supplement: S1 Fig — Western blot analysis of STAT3, p-STAT3, Akt, p-Akt and CCK-BR protein levels in the xenograft tumor tissues of NCI-N87/miR-148a, NCI-N87/nc and NCI-N87 groups. (TIF) [file pone.0158961.s001.tif]

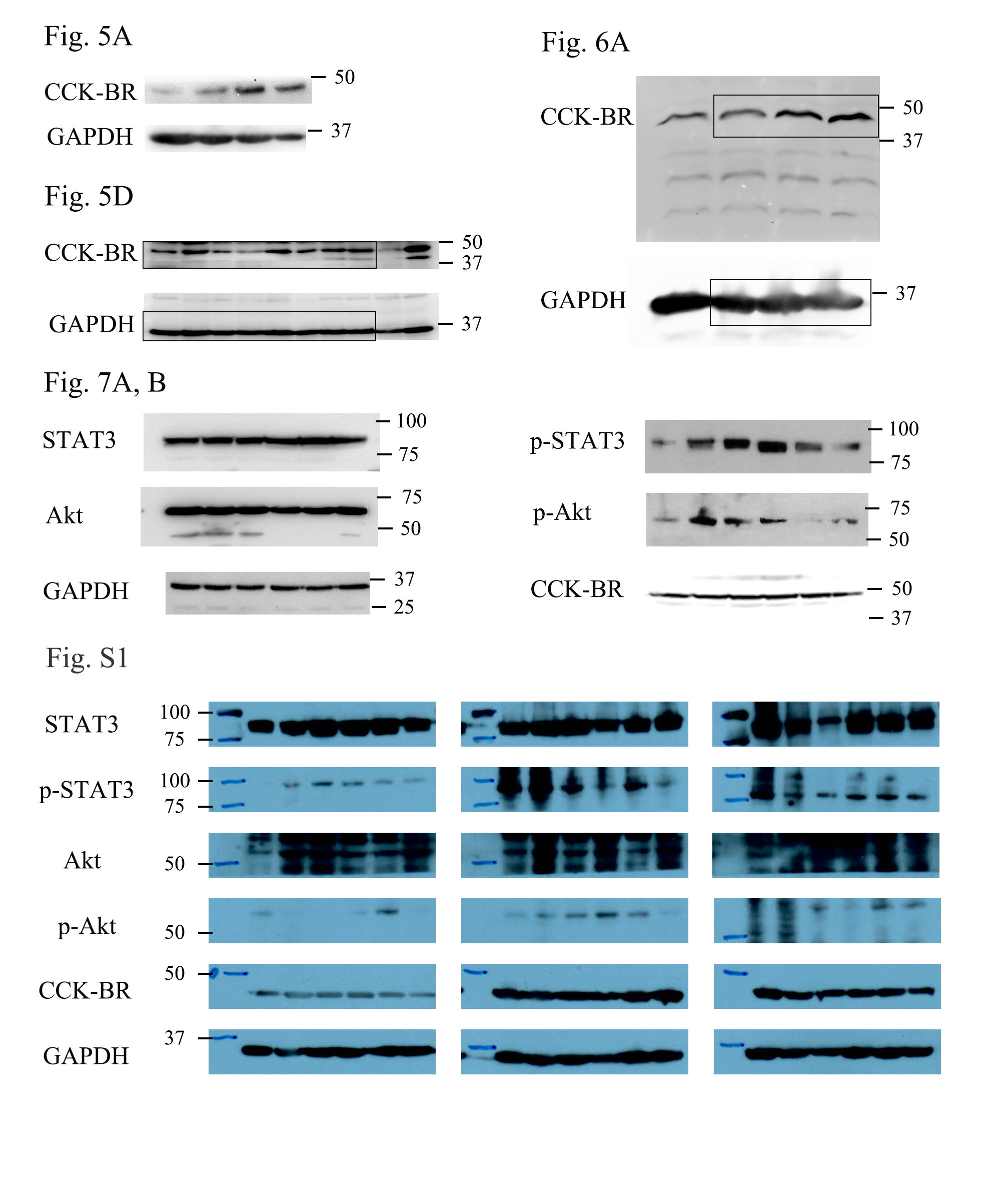

Supplement: S2 Fig — (TIF) [file pone.0158961.s002.tif]
